# A Bioartificial Renal Tubule Device Embedding Human Renal Stem/Progenitor Cells

**DOI:** 10.1371/journal.pone.0087496

**Published:** 2014-01-30

**Authors:** Anna Giovanna Sciancalepore, Fabio Sallustio, Salvatore Girardo, Laura Gioia Passione, Andrea Camposeo, Elisa Mele, Mirella Di Lorenzo, Vincenzo Costantino, Francesco Paolo Schena, Dario Pisignano

**Affiliations:** 1 Center for Biomolecular Nanotechnologies, Istituto Italiano di Tecnologia, Arnesano, Italy; 2 Nephrology, Dialysis and Transplantation Unit, Department of Emergency and Organ Transplantation, University of Bari, Bari, Italy; 3 Centro Addestramento Ricerca Scientifica in Oncologia (C.A.R.S.O.) Consortium, Valenzano, Italy; 4 Department of Science, Biological and Environmental Sciences and Technologies, University of Salento, Lecce, Italy; 5 National Nanotechnology Laboratory of Istituto Nanoscienze-CNR, Lecce, Italy; 6 Dipartimento di Matematica e Fisica “Ennio De Giorgi”, Universitá del Salento, Lecce, Italy; University of Torino, Italy

## Abstract

We present a bio-inspired renal microdevice that resembles the *in vivo* structure of a kidney proximal tubule. For the first time, a population of tubular adult renal stem/progenitor cells (ARPCs) was embedded into a microsystem to create a bioengineered renal tubule. These cells have both multipotent differentiation abilities and an extraordinary capacity for injured renal cell regeneration. Therefore, ARPCs may be considered a promising tool for promoting regenerative processes in the kidney to treat acute and chronic renal injury. Here ARPCs were grown to confluence and exposed to a laminar fluid shear stress into the chip, in order to induce a functional cell polarization. Exposing ARPCs to fluid shear stress in the chip led the aquaporin-2 transporter to localize at their apical region and the Na^+^K^+^ATPase pump at their basolateral portion, in contrast to statically cultured ARPCs. A recovery of urea and creatinine of (20±5)% and (13±5)%, respectively, was obtained by the device. The microengineered biochip here-proposed might be an innovative “lab-on-a-chip” platform to investigate *in vitro* ARPCs behaviour or to test drugs for therapeutic and toxicological responses.

## Introduction

The human body is a heterogeneous and perfectly synchronized system, composed of different organs that are in turn made up of several, small and functionally autonomous units, called micro-organoids, such as lobuli in the liver, nephrons in the kidney and alveoli in the lung. The behavior of a single micro-organoid is considered representative of the whole organ functionality [1]. For an in-depth understanding of human physiology and for promoting advances in medicine and toxicology, the availability of engineered platforms able to reproduce functional portions of living organs is challenging [2].

In this framework, a useful tool is offered by microfluidic techniques [3]–[5], namely of devices for cell culture that closely mimic physiological aspects of a well-organized biosystem at the same micro-scale as living cellular milieu [6]–[8]. Differently from standard culture systems, microfluidic devices provide a tight control over flow conditions [5], [9], and the distinctive possibility of maintaining constant fluid perfusion inside microchannels [10] to induce a shear stress, which is advantageous for the functionality of many cells, including renal tubular cells [11], [12]. A recent advance enabled by the microfluidic approach consists in the fabrication of engineered “organs-on-a-chip” [13], re-creating *in vitro* micro-compartments of blood vessels [14], [15], liver [16], [17], brain [18], gut [19] and lung [20]. The aim of these studies is to reproduce the structural arrangements and biological functions of micro-organoids.

A critical issue, in this context, is the cell source to be used in designing and developing organs-on-chip. Immortalized cell lines are very common and well characterized, but they show considerable phenotypic and genetic divergences if compared with *in vivo* human cells. Primary cell lines do not present this inconvenience but are scarcely available and difficult to culture over a long period of time. The use of adult stem cells extracted from patients would overcome these difficulties. In the kidney, resident adult renal stem/progenitor cells (ARPCs) have been identified [21], [22], raising a lot of interest due to their potential therapeutic applications [23]–[25]. These cells, isolated both from the tubule interstitium [24] and Bowman’s capsule [25], showed multipotent differentiation properties, by generating tubular epithelial-like, osteogenic-like, adipocyte-like and neuronal-like cells [21], [22], [26]. *In vitro*, they can differentiate into epithelial cells expressing some markers of renal proximal and distal epithelium. *In vivo*, these cells can express some markers of both distal tubules such as the NaCl co-transporter and calbindin-D of proximal tubules [25]. After the injection into mice with acute kidney injury, ARPCs showed an exceptional regenerating capacity for injured renal tubular cells and a marked resistance to apoptotic events [24], [27], [28]. For these peculiar abilities, ARPCs may be considered the future direction of renal regenerative medicine and a promising tool to treat acute and chronic renal injury [27]–[30].

Here we present a bioartificial proximal tubule-like structure based on a multi-layer microdevice embedding ARPCs. The device was composed of two overlapped elastomeric layers, sandwiching a porous polycarbonate (PC) membrane. The geometry was purposely designed to mimic the *in vivo* structure of a renal tubule, with the upper microchannel providing the lumen area, in which the apical portion of cells was exposed, and the lower microchannel simulating the interstitial area in contact with the basolateral membranes of cells. According to the design of a bioartificial renal tubule [31], living cells were seeded on the polymeric membrane, which was water and solute permeable, to ensure the transport of solutes across it, and acted as scaffold for cell growth [32]. Biochemical and physical parameters were optimized and used to promote the on-chip confluent growth of ARPCs, which were then exposed to physiological laminar fluid shear stress (FSS) and characterized for their recovery of urea and creatinine, analyzing the fluid outlets collected from the device. The induction of cell polarity in ARPCs was well characterized with apical and basolateral marker proteins, thus demonstrating that the feasibility of renal tubules-on-chip may open new perspectives also in view of the parallelization and adjuvant therapy for renal failure.

## Materials and Methods

### Materials

The poly(dimethylsiloxane) (PDMS) elastomer (Sylgard 184 Silicone Elastomer Kit) was purchased from Dow Corning (Midland, MI). Capillary tubing connections (Tygon), with an inner diameter (i.d.) of 0.5 mm and outer diameter (o.d.) of 1.5 mm, were from Norton Performance Plastics (Akron, OH). SU-8 for master fabrication was purchased from MicroChem Corp. (Newton, MA). The Nuclepore™ track-etched PC membrane (porosity size 0.1 µm, thickness 40 µm) was purchased from Whatman (Kent, United Kingdom). CellCrown™ Cell Culture Inserts were purchased from Scaffdex Oy (Tampere, Finland). Rhodamine 6G dye (R6G 99%, molecular weight of 480 Da), Dulbecco’s Modified Eagle’s Medium Mixture F-12 Ham (DMEM/F12), fetal bovine serum (FBS), phosphate buffered saline (PBS), Hank’s Balanced Salt Solution modified (HBSS-CMF), 0.05% trypsin/0.2% ethylenediaminetetracetic acid (EDTA) solution, penicilline-streptomycine solution (10^5^ units of penicillin/mL and 10 mg streptomycin/mL), Triton® X100, bovine serum albumin (BSA), 4′,6-diamidino-2-phenylindole dihydrochloride (DAPI), phalloidin–Tetramethylrhodamine B isothiocyanate (phalloidin-TRITC), bovine plasma fibronectin, laminin, anti-fibronectin produced in rabbit antibody, Fluorescein Isothiocyanate (FITC)-labelled anti-rabbit IgG antibody, glucose (GO) assay kit and organic solvents were purchased from Sigma-Aldrich (St. Louis, MO). The basement membrane matrix BD Matrigel™ was from BD Bioscience (Franklin Lakes, NJ). Microvascular Endothelial cell Growth Medium (EGM-MV) and Renal Cell Growth Medium (REGM) bullet kits were purchased from Lonza (Milan, Italy). Cell-Titer 96 AQueous One Solution Cell Proliferation Assay (MTS) was from Promega (Milan, Italy). CD133Ab-conjugated magnetic microbeads were purchased by Miltenyi Biotec, Bergisch Gladbach, Germany. The antibodies PE-conjugated anti-CD133/2 (293C3), FITC-conjugated anti-CD34, FITC-conjugated anti-CD45, mouse anti-human CD133/1 mAb (clone AC133) and mouse antihuman CD133/2 mAb and FcR blocking reagent were from Miltenyi Biotec. FITC-conjugated anti-CD105 and FITC-conjugated anti-CD24 and FITC-conjugated mouse IgG1 were from Serotec (Oxford, UK). FITC-conjugated anti-CD44 was from Instrumentation Laboratory (Milan, Italy). Rabbit anti-human Pax-2 pAb was from Covance (Princeton, NJ), mouse anti-human CD105 mAb and rabbit anti-human Oct-4 pAb were from Abcam (Cambridge, UK), mouse anti-human CD24 mAb was from Dako (Glostrup, Denmark), mouse anti-human CD44 mAb was from Chemicon (Temecula, CA), mouse anti-human Bmi1 mAb was from Upstate Biotechnology (Lake Placid, NY). The secondary antibodies Alexa Fluor 555 goat anti-mouse IgG, Alexa Fluor 488 goat anti-rabbit IgG, Alexa Fluor 488 goat anti-mouse IgG1 and the dye To-pro-3 were from Molecular Probes (Eugene, OR). The antibodies Na^+^/K^+^ ATPase, aquaporin-2 (AQP2) and Zonula occludens 1 (ZO-1) were provided by Santa Cruz Biotechnology (Santa Cruz, CA). The Gel/Mount for immunofluorescent characterization was from Biomeda (Milan, Italy). Cells from human renal proximal tubule epithelial cell line (human kidney-2, HK-2) [33] were provided by the CARSO consortium bio-bank chaired by Prof. F.P. Schena. Renal Proximal Tubule Epithelial Cells (RPTECs) were purchased from Lonza (Lonza, Basel, Switzerland). The creatinine and urea dosage kits were from SGM Italia (Rome, Italy).

### Device Fabrication and Characterization

The device core was a porous PC membrane sandwiched between two PDMS layers, each having an engraved microchannel with a width of 500 µm and a height of 120 µm. The master structures used for PDMS replicas were fabricated by photolithography (UV exposure for 10 s at 500 W) on SU-8 2100 photoresist. The resist was spin-cast (3000 rpm, 30 s) on Si/SiO_2_ substrate and developed for 8 min after the bake processes (65°C for 5 min and 95°C for 30 min). The elastomeric layers were realized by replica molding through *in situ* polymerization (75°C for 15 min) of PDMS (10∶1 w/w base: curing agent) on the master, peeled off and punched in correspondence to the inlet and outlet chambers of the microchannels. In lieu of performing oxygen plasma treatments [11], the PC membrane was embedded between the two PDMS structured layers by introducing a thin mortar layer [34] that was prepared by spin-coating a mixture of PDMS and toluene (40% w/v) on a clean glass cover slide (1500 rpm for 1 min). Then, the two PDMS elements were placed onto the glass slide coated with the adhesive mortar layer and allowed to stay in contact for 30 s. Finally, the PC membrane was placed onto one of the PDMS layers and pressed down into the thin adhesive film. This PDMS piece with the membrane attached was then aligned under a stereomicroscope and bonded with the second PDMS layer through overnight thermal curing at 35°C, which avoided shrinkage of the membrane.

The membrane-integrating device was connected for fluid injection and outlet collection through plastic tubes that were fitted into the inlet and outlet ports. The extremities of the tubes were coupled to the stainless steel needles of 2.5 mL syringes (inlets), connected to an infusion pump (Harvard Apparatus, Holliston, MA) and to eppendorf tubes (outlets), respectively.

Traps were inserted in the microfluidic circuit between needles and inlets to prevent air bubble formation in the perfusion cell culture. The bubble traps were constructed by bonding a flat and a textured layer of PDMS with oxygen plasma (50 W for 15 s). The structured element was fabricated by replica molding (75°C, 15 min) starting with an SU-8 master made up of two continuous straight channels (width 500 µm, height 120 µm), inlet and outlet, connected by a circular ridge (radius 4 mm, height 120 µm) on the top of which was placed a metal spacer (radius 4 mm, height 1 cm). For producing the flat elastomeric layer, the PDMS was spin-cast (1500 rpm for 30 min) on a glass substrate and cured (75°C, 15 min). When an air bubble approached the circular trapping chamber, it was captured and ruptured upon reaching the liquid-air interface.

To analyze the device functionality in terms of diffusion of molecules through the porous membrane, preliminary diffusion tests were carried out by fluxing, in counter-current mode, a solution of rhodamine 6G (1 mg mL^−1^) and distilled water into the two microchannels, respectively, at a flow rate of 1 µL min^−1^. Fluorescence variations were recorded with a microscope (Leica MZ16FA) equipped with a fluorescent lamp (100 W) and a video camera (Leica DFC490), and micrographs were analyzed by the ImageJ software. As control, rhodamine diffusion tests were also performed on a standard system, composed by the PC membrane placed in a CellCrown™ Cell Culture Insert and positioned in a 12-well polystyrene plate. The lower compartment was filled with distilled water and the upper compartment with rhodamine 6G solution (1 mg mL^−1^). The fluorescence variation in the lower compartment was recorded with an inverted microscope Eclipse Ti equipped by Nikon confocal A1 R MP system. 400 frames per second were analyzed by the Nikon NIS Element software.

### HK-2 and RPTEC Cell Culture

First, the device parameters were optimized to obtain an on-chip confluent monolayer of human kidney HK-2 cells [33], used as the model cell type for renal tubule cells. To this aim, HK-2 cells were cultured in the presence of the DMEM/F12 medium supplemented with 10% FBS and 1% penicilline-streptomycine. RPTECs were cultured in the recommended medium REGM. Both cell lines were sub-cultured at least once a week and maintained at 37°C under a humidified atmosphere, constituted by 95% air and 5% CO_2_.

### ARPC Isolation and Cell Culture

ARPCs were obtained from fresh human renal cortical tissues harvested from patients with localized renal cell carcinoma that underwent nephrectomy, according to standard clinical protocols. At the time of radical nephrectomy, all patients gave signed consent for the use of part of removed tissues for research purposes. Portions of normal-appearing cortex were isolated surgically and histologically examined to exclude the presence of carcinoma. The CD133-positive ARPCs were isolated and characterized as previously described [26], [28], [35], [36]. Briefly, the cortex renal fractions were dissected by passage through a graded series of steel meshes sieves to remove the fibrous component. The cellular fraction was then passed through a 120-mesh sieve to isolate the capsulated glomeruli from the tubular fraction. After several washes, the two isolated fractions were cultured separately in the EGM-MV medium supplemented with 20% FBS. After 4–5 days, the cultures were washed twice with PBS buffer to remove non-adherent cells and after about 1 week in culture cell viability and number were checked. The CD133-positive cells were then isolated by magnetic cell separation technology (MACS) by means of CD133Ab-conjugated magnetic microbeads. The eluted cells were resuspended and maintained in the EGM-MV medium supplemented with 20% FBS and incubated at 37°C with 5.0% CO_2_.

The ARPCs markers were checked by cytofluorimetric determination and by cell immunofluorescence microscopy. Cytofluorimetric assays were performed using a Partec Flow-Max cytofluorimeter (Munster, Germany) and the following primary human antibodies: anti-CD133/2 (293C3), anti-CD34, anti-CD45, anti-CD105, anti-CD24 and anti-CD44. FITC-conjugated mouse IgG1 was used as an isotype control. Non-specific sites were blocked with the FcR blocking reagent. Each determination was performed on 10^5^ cells.

For immunofluorescence experiments, ARPCs were fixed in 4% paraformaldehyde. The cells were blocked for 1 h (BSA in PBS, pH 7.4) and then incubated with monoclonal or polyclonal primary antibodies overnight at 4°C or for 2 h at room temperature, respectively. The following primary human antibodies were used: anti-CD133/1 mAb, anti-CD133/2 mAb, anti-Pax-2 pAb, anti-CD105 mAb, anti-CD24 mAb, anti-CD44 mAb, anti-Bmi1 mAb, anti-Oct-4 pAb and ZO-1. The immune complexes were identified after the incubation of the cells with the specific secondary antibodies for 1 h at room temperature. The cells were washed in PBS after each step, counterstained with To-pro-3, mounted in Gel/Mount, and sealed with nail varnish. The stained cells were viewed under the Leica TCS SP2 (Leica, Wetzlar, Germany) confocal laser-scanning microscope.

### Membrane Surface Treatment

Before seeding cells in the device, extensive tests were carried out to choose the most suitable extracellular matrix (ECM) protein for the biofunctionalization of the PC membrane. Three different surface treatments were analyzed: fibronectin (10 µg/mL in PBS), laminin (10 µg/mL in PBS) and Matrigel (a dilution 1∶5 in complete DMEM/F12 culture medium). The PC membranes, placed in the 6-well polystyrene plates, were covered with a volume of 0.5 mL of each protein solution, and incubated at 4°C for 2 h. Confluent HK-2 cells in the flask were then washed with PBS, removed with a trypsin/EDTA solution and seeded on functionalized membranes at a concentration of 1×10^5^ cells mL^−1^. As controls, cells were also seeded on untreated membranes and polystyrene wells. Cell viability on all tested substrates was evaluated after 2 days by MTS assay, by exploiting the conversion of tetrazolium salt to soluble formazan dye due to metabolically active cells. Experiments on each set of samples were repeated three times.

The apparent static water contact angle (WCA, θ) of the bare and treated membrane was also investigated, by means of a KSVCAM200 instrument (KSV, Finland). Distilled water droplets with a typical volume of about 2 µL were dispensed onto the surfaces by a syringe, connected to the contact angle measuring system.

### Cell Seeding

Before each experiment, the chip and all the fluidic connectors were sterilized by UV germicidal irradiation (8 W lamp, G30T8, Sankyo Denki) in a laminar flow hood for 1 h. For cell seeding, the same procedure was carried out for HK-2, RPTECs and ARPCs. Briefly, confluent cells in the flask were washed with HBSS, removed with a trypsin/EDTA solution and seeded in the fibronectin-coated device at typical concentrations of 0.5 and 1.5×10^6^ cells mL^−1^. After 24 h, non-attached or dead cells were removed by rinsing in the cell culture medium. For static culture devices, both HK-2 and ARPCs were grown to confluence on fibronectin-coated membranes over 4 days after seeding in a CO_2_ incubator by entirely changing the culture medium every 8 h. In this way the supply of the fresh cell medium and the removal of the metabolic waste were both ensured. In order to investigate the ARPC morphology in macroscopic cultures, cells were seeded on standard systems composed by the porous PC membrane placed in a cell culture insert. In detail, after coating the surface of the membrane with fibronectin (10 µg/mL in PBS), a concentration of 1.5×10^6^ cells mL^−1^ was transferred onto the membrane. Also in these experiments, cells were grown over 4 days after seeding in a CO_2_ incubator by changing the culture medium every 8 h. As negative controls, microfluidic devices without cells were stored in a CO_2_ incubator under identical conditions and undergoing the same medium changes. Experiments on each set of samples were repeated three times, on different devices.

### Flow Tests

Once cell confluence onto the membrane surface was reached, the lumen microchannel was perfused with the complete culture medium supplemented with urea (25 mg dL^−1^), creatinine (1 mg dL^−1^) and glucose (0.01 mg dL^−1^); the interstitial microchannel was instead fluxed with the complete culture medium without urea and creatinine and glucose in counter-current with respect to the lumen flow. Cells were exposed to a volumetric flux of 1 µL min^−1^ for 6 h at 37°C. Outlet samples from both the lumen and interstitial microchannels were taken and the recovery of urea, creatinine and glucose across the porous membrane colonized by cells was evaluated, following well-established colorimetric methods. Microchips without cells were analyzed as negative controls. Experiments on each set of samples were repeated three times on different devices.

### On-chip Immunofluorescence Assays

After completing flow measurements, immunocytochemistry experiments were performed in order to assess the morphology and growth of renal tubule cells within the device. Staining was carried out directly in the chip, rinsing with PBS, fixing cells grown on the PC membrane by a solution of 4% paraformaldehyde in PBS for 20 min, and washing 3 times with PBS for 5 min each. Cell membranes were permeabilized by incubation with 0.1% (v/v) Triton-X100 in PBS for 10 min, followed by incubation in 1% BSA in PBS for 30 min to reduce nonspecific background staining. For investigating cell morphology, the device was incubated for 40 min with phalloidin-FITC/TRITC (25 µg/mL), washed with PBS and stained with DAPI (3 µg/mL) for 10 min. To visualize the markers AQP2, Na^+^K^+^ATPase, before phalloidin and DAPI staining, chips were incubated for 2 h in the primary antibody (anti-AQP2 1:50 in BSA; anti- Na^+^K^+^ATPase 1∶50 in BSA), washed with PBS and incubated with the secondary antibody (anti goat-FITC 1∶100 for AQP2 staining; anti mouse–FITC 1∶400 for Na^+^K^+^ATPase staining) for 1 h. Finally, the membrane, with cell attached, was taken apart from the device and visualized by inverted microscopy Eclipse Ti equipped by confocal A1 R MP system (Nikon, Melville, NY). Experiments were repeated three times on different devices.

## Results

### Design and Characterization of the Microfluidic Device

**Figure 1:**
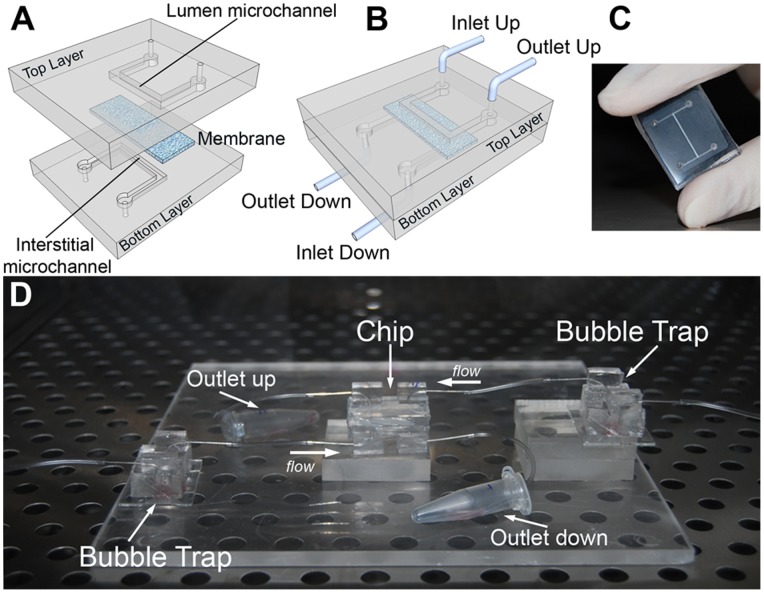
The bio-inspired renal microdevice. The multi-layered chip, resembling the in vivo structure of a proximal kidney tubule, was composed by two overlapped PDMS layers (A), with engraved microchannels. The two microchannels simulated the lumen area in which the apical portion of cells is exposed (upper channel) and the interstitial area in contact with basolateral membranes (lower channel). The channels were physically separated by a microporous PC membrane and were continuously fed in counter-current modality. Once assembled (B), the device presented two inlet and two outlet ports, each connected to plastic tubes and syringe pumps for fluid control. (C) Photograph of the bioartificial proximal tubule microfluidic device. (D) Complete experimental set-up including the chip and distinct bubble traps for the inlet ports.

In our bioartificial proximal tubule, shown in Fig. 1A–C, both the top and the bottom layers were made of PDMS, chosen for its well-established oxygen permeability, ease of use and optical transparency [9], [20]. While PDMS has been used in many bioengineered microfluidic devices [11], in our report each PDMS layer contained an engraved microchannel coupled to external flows. This architecture supported two different fluid streams, separated by a porous membrane colonized by cells. The PC membrane is highly compatible with proteins and cells, and exhibits an optimal ultrafiltration capacity and a very low risk of solute back diffusion [37]. The inlet and outlet ports of the device were connected to plastic tubes and syringe pumps, for controlling the fluid passage in each microchannel independently (Fig. 1D). Finally, the device was connected with two bubble traps (Fig. 1D) to prevent the entry of air bubbles and ensuring the viability of cells cultured in the microchip. In fact, when an air bubble approaches the cells, these can be stretched by forces exerted at the liquid-air interface, which may lead to the rupture of the cell membranes [38].

**Figure 2:**
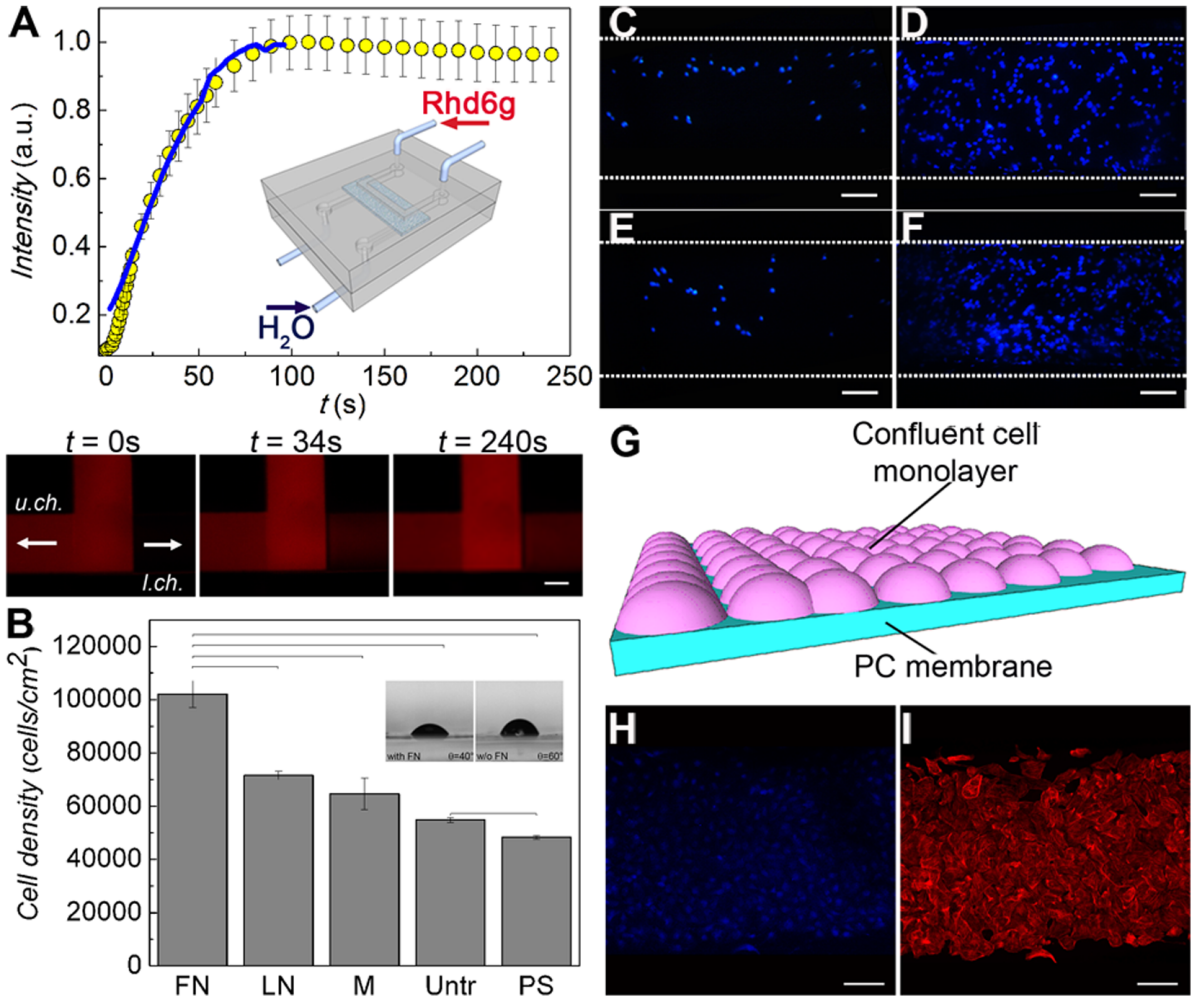
On-chip formation of a confluent monolayer of renal tubule cells. (A) Device fluidic characterization by diffusion test: a solution of rhodamine 6G was fed in the upper channel while distilled water was fed in the lower channel (interstitial). The increase in the fluorescence intensity with time at the outlet of the lower channel was correlated to the rhodamine diffusion into the channel. The superimposed line is the corresponding data in a standard cell culture insert using the same porous membrane. Inset: device and counter-current flow schematics. (B) HK-2 cell proliferation described by MTS assay after 2 days of culture on the membranes functionalized with fibronectin (FN), laminin (LN) and Matrigel (M), compared to the untreated membranes (Untr.) and the positive controls of polystyrene dishes (PS). Results are expressed as (mean ± standard deviation) of three *independent experiments*. Bars show statistically significant differences (P<0.05). (*inset*) Optical micrographs of water droplets on membranes with or without FN, and corresponding WCA value. (C–F) Optimization of HK-2 cell growth in the device. A starting concentration of 5×10^5^ cells mL^−1^ was insufficient for a successful colonization of the membrane by cells (C), while a concentration ≥ 1.5×10^6^ cells mL^−1^ led to a confluent growth (D). Culturing cells for 4 days under a constant flux (1 µL min^−1^) of cell medium did not allow the formation of a continuous monolayer (E). Cell confluence was instead achieved by letting seeded cells in a static fluid environment for 24 h and culturing them over 4 days by changing the complete growth medium twice a day (F). Scale bars = 100 µm. (G) Scheme of the resulting cell confluent monolayer on the membrane. Confluent living cells were stained with DAPI (blue) (H) and TRITC-phalloidin (red) (I). Scale bars = 100 µm.

Firstly, the microfluidic device was characterized by dye-diffusion through the PC membrane. Rhodamine is a good dye system in this respect for its bright fluorescence and because its hydrodynamic radius (7.7×10^−10^ m) is comparable to those of solutes involved in kidney functions such as urea (1.8×10^−10^ m) and creatinine (2.6×10^−10^ m). Experiments were carried out by fluxing, in counter-current mode, a rhodamine 6G solution (1 mg mL^−1^) and distilled water into the two microchannels, respectively. The resulting temporal curve represented the mean of three independent experiments, carried out on different devices. As shown in Fig. 2A, the fluorescence intensity grew with time at the outlet of the lower channel, corresponding to an increase of rhodamine 6G concentration. The diffusion process stabilized in a time, *τ_S_*, of 100 s, necessary to bring the device to the stationary conditions to be cleared (Fig. 2A). The temporal behaviour of the increase of the fluorescence intensity was found to be basically the same as in a system where two compartments were separated by the same membrane used in the microfluidic device, and under static conditions of the liquids (superimposed line in Fig. 2a). This indicated that diffusion was indeed the dominant mechanism leading molecules to pass through the pores, instead of leakages and of possible contributions from convection, which in principle could occur in counter-flow modality due to the presence of pressure differences in the microchannels on the two sides of the membranes [39]. In addition, the apparent diffusion coefficient across the membrane, *D_m_* ≅ *d* ^2^/*τ_S_*, where *d* is the membrane thickness, was of 16 µm^2^/s lower than the diffusion coefficient of rhodamine in free water (414 µm^2^/s) [40]. The corresponding membrane permeability or mass transfer coefficient, *K* = *D_m_*/*d*, was about 0.40 µm/s for the here used fluorescent compound (molecular weight of 480 Da), in good agreement with the value (0.34 µm/s) found for other rhodamine species (sulforhodamine, 607 Da) moving across a similar, 6 µm-thick PC membrane [41].

### Functional Renal Biochip

To choose the most suitable ECM protein for the functionalization of the PC membrane, the proliferation of HK-2 cells on PC membranes coated with either fibronectin, laminin or Matrigel was compared. Untreated membranes and polystyrene substrates were included as control (Fig. 2B). The number of metabolically active cells on fibronectin-coated membranes (∼ 10^5^ cells) was about 1.4 and 1.6 times higher than that obtained by membranes functionalized with laminin and Matrigel, respectively. Untreated membranes and polystyrene substrates showed a proliferation rate about two times lower. In particular, we noticed that even the untreated membranes were a more suitable scaffold than standard polystyrene surfaces, due to the microporous structure that mimics the tridimensional microarchitecture of native ECM [42]. The wetting properties of membranes were also investigated by contact angle measurements. The functionalization by fibronectin led to a decrease of the WCA by about 20° (Fig. 2B), thus enhancing surface wettability, which is known to favour cell adhesion, spreading and growth [43]. The on-chip investigation by immunostaining of the fibronectin coating finally demonstrated a roughly uniform distribution of the protein along the microchannel (Fig. S1).

Then we optimized the culture conditions on-chip by using HK-2 cells as model. An initial concentration of 1.5×10^6^ cells mL^−1^ was found to effectively lead to cell confluence onto the membrane (Fig. 2C, D). Furthermore, dynamic and static flow conditions were compared (Fig. 2E, F). In the first case, the system was continuously fed for 4 days at a constant flow rate of 1 µL min^−1^ after cell seeding. However, the flux might have interfered with the cell adhesion onto the membrane since a continuous monolayer of cell was not observed (Fig. 2E). On the other hand, the confluence was achieved (Fig. 2F, 2G) when, after seeding, the cells grew under a static culture environment for about 24 h, followed by changes of the culture medium twice a day.

Once the device architecture and working conditions were established, we realized the functional renal tubule biochip. After 4 days of cell culture, the lumen microchannel was perfused with the complete culture medium containing urea (25 mg dL^−1^) and creatinine (1 mg dL^−1^) and the same solution (but without urea and creatinine) was injected into the interstitial microchannel in counter-current mode. Flow rates were chosen to mimic physiological FSS to which renal cells are exposed, ranging between 0.2 and 20 dyn cm^−2^ [44], [45]. The FSS produced at the microchannel walls can be estimated by *τ = *6 *µQ/bh* ^2^, where *μ* is the medium kinetic viscosity (dynes cm^−2^), *Q* is the volumetric flow rate, *b* is the channel width, and *h* is the channel height [11], [44]–[46]. In our case, a volumetric flux of 1 µL min^−1^ corresponded to a FSS of about 0.2 dyn cm^−2^, applied for 6 h after waiting about 100 sec to reach the stationary conditions. The use of FSS has been demonstrated to be important for the functional behaviour of these cells [47], since it can regulate the formation of tight junctions, the regulation of ion movements and the homeostasis of water [44], [45].

Outlet samples from both lumen and interstitial microchannels were collected to quantify the percentage recovery of urea and creatinine, calculated as:
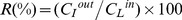
(1)where 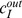
is the output solute concentration collected from the interstitial channel and 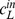 is the input solute concentration in the lumen channel. In a counter-current arrangement, the two solutions flow in opposite directions, and at any coordinate nearby the membrane the solute concentration in the interstitial channel is lower than that in the lumen channel [39]. Hence, the recovery can be higher than 50%. Our device employed a counter-flow arrangement, as in renal tubules *in vivo*, with equal flow rates in the two channels. The results, expressed as (mean ± standard error), were obtained from three independent experiments performed on different devices. For devices with cells, we obtained a recovery of (16±5)% for urea and (18±5)% for creatinine. In control experiments without cells, performed on fibronectin-coated membranes, the values increased to (64±7)% and (45±7)%, respectively, indicating a higher overall permeability in absence of cellular coverage on the membrane surface (Fig. 2G). For sake of comparison, we recall that a recovery up to about 80% has been reported for glucose diffusing through a PC membrane separating a static reservoir and a channel supporting a 1.5 µL min^−1^ flow [41]. Here, the presence of a highly uniform layer of HK-2 cells in the bioartificial device was also confirmed by immunocytochemistry assays performed to stain nuclei labeled with DAPI (blue fluorescence) (Fig. 2H) and actin filaments with phalloidin-TRITC (red fluorescence) (Fig. 2I).

### Stem Cell Isolation

To reproduce the essential functions of a living kidney, it is necessary to replace standard immortalized cell lines with primary renal human cells. A possible strategy could be, in principle, the use of primary cells which are phenotypically and physiologically similar to *in vivo* human cells. For instance, RPTECs, which are primary human renal proximal tubule epithelial cells, are commonly used as model system for kidney cell biology [48]. However, we found that RPTECs did not colonize the microchip appreciably, regardless of the seeding concentration (Fig. S2). This result, attributable to the much higher sensitivity of these cells to culture conditions, with respect to immortalized cell lines, strengthens the importance of the use of stem cells in kidney-mimicking bioartificial chips.

**Figure 3:**
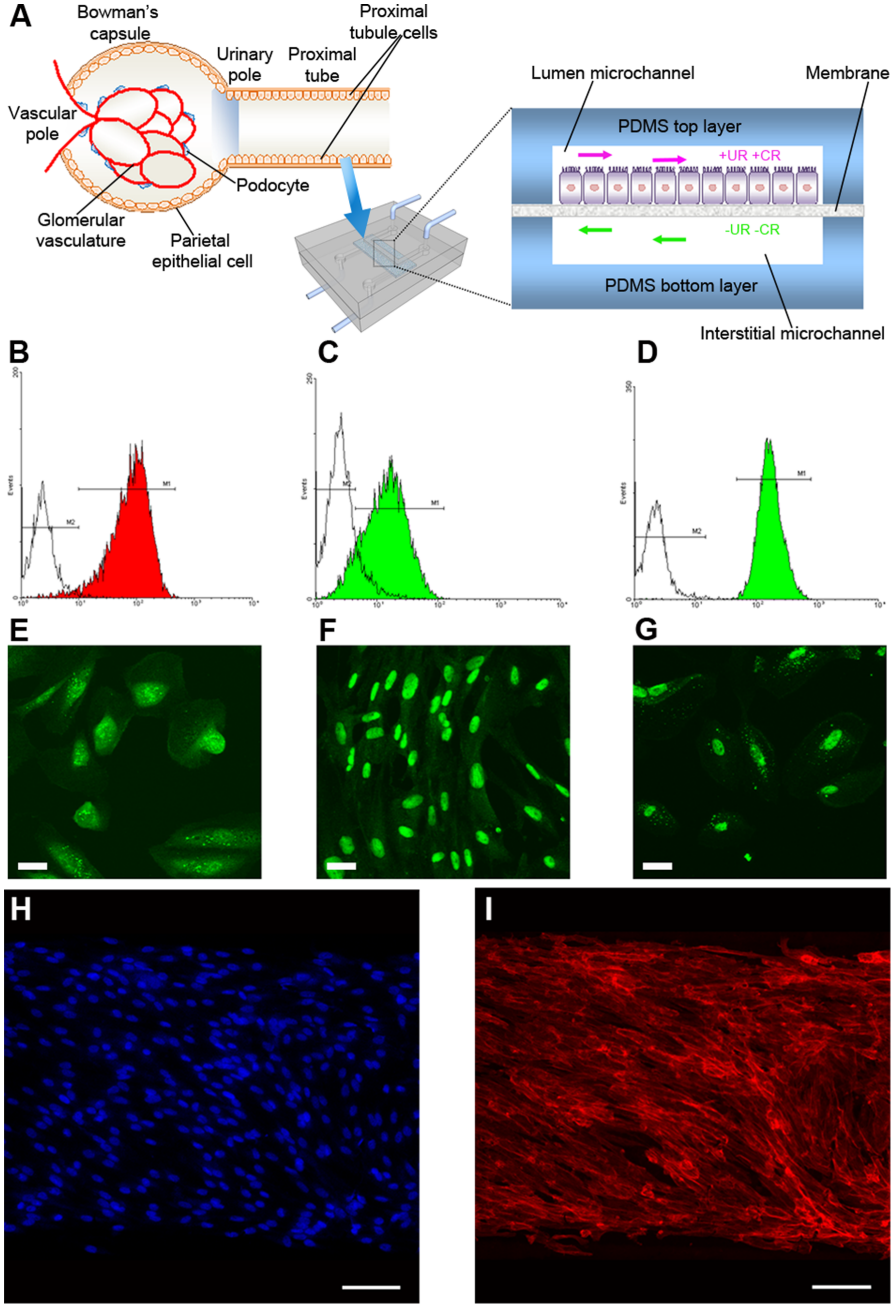
Fulfillment of a bioartificial proximal tubule on-a-chip embedding ARPCs. (A) Scheme of the glomerulus and the proximal tubule structure in a human kidney nephron. Here tubular ARPCs were seeded into the device, whose cross section illustrates a confluent layer of ARPCs within the lumen microchannel and adherent to the membrane. After 4 days of culture, the lumen microchannel was perfused with the complete culture medium containing urea (UR) and creatinine (CR) and the medium without UR and CR was injected in counter-current into the lower, interstitial microchannel. (B–G) Characterization of isolated tubular ARPCs. Cytofluorimetric analysis shows the expression of CD133 (B), CD24 (C), CD44 (D). Immunofluorescence detection evidences the expression of Oct-4 (E), PAX-2 (F), BMI-1 (G). Scale bars = 50 µm. (H–I) Confluent growth of ARPCs in the device attested by immunostaining of cells with DAPI (blue) (H) and TRITC-phalloidin (red) (I). Scale bars = 100 µm.

Differently from RPTECs, ARPCs indeed retain a greater plasticity [49], [50] and exceptional adaptability to various microenvironments. We firstly extracted ARPCs from the tubular portion of the cortex renal fraction, in order to realize a reliable bioartificial proximal tubule-like microdevice based on a multi-layer microdevice (Fig. 3A). Therefore, we characterized tubular ARPCs for renal stem cell markers by confocal microscopy and fluorescence-activated cell sorting (FACS) analysis. Our recovered populations were homogeneously positive for stem cell markers CD133 and CD24 and for the renal transcription factor PAX-2 (Fig. 3B, C and F). However, the CD34, CD105, and CD45 markers of mesenchymal and hematopoietic stem cells were not detectable (data not shown), thus demonstrating that we were dealing with ARPCs resident in the kidney. Moreover, these CD133+ cells expressed the hyaluronic acid receptor CD44, the blastocyst stem cell marker Oct-4, and the adult stem cell marker BMI-1 (Fig. 3D, E and G, respectively), all of which are typically expressed in ARPCs [25], [26]. CD133+ CD24+ ARPCs can be maintained in culture without losing CD133 expression for up to 7–9 passages [24], giving rise to homogenous clonal populations [26]. Moreover, renal progenitors can grow continuously for 60 to 90 population doublings, depending on the donor, during a period of 4 months and when assessed at 50 population doublings, cells exhibited diploid DNA content [25].

### Stem Cell Growth and Polarization on Chip

ARPCs in the chip were cultured under the conditions previously described for HK-2 cells. After 4 days, solute diffusion experiments evidenced a recovery of urea and creatinine of (20±5)% and (13±5)%, respectively, which were comparable with values obtained by HK-2 cultures. This was in agreement with the formation of a layer of stem cells on the microporous membrane [51], [52]. We also measured the recovery of glucose, which was found to be of (52±5)% in the ARPC-based device.

**Figure 4:**
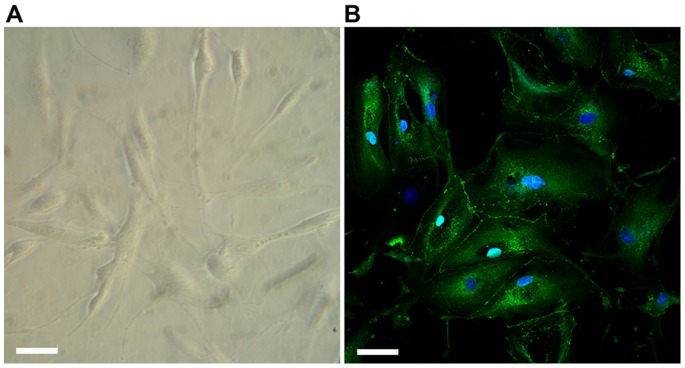
Characterization of ARPC morphology. (A) Light microscopy image of ARPCs. (B) ARPCs can be differentiated in renal tubular cells, and form junctions as shown by ZO-1 immunostaining (rabbit anti-human ZO-1 polyclonal Ab, green). To-pro-3 counterstains nuclei (blue). Scale bar = 40 µm.

In order to assess the morphology and the cytoskeleton organization of stem cells in the bioartificial device, we performed immunocytochemistry assays by staining actin filaments labeled with phalloidin-TRITC (red fluorescence), and nuclei with DAPI (blue fluorescence), which confirmed that the polymeric membranes were uniformly covered (Fig. 3H, I). Cells grew strictly confined along the tubule-like microchannel without penetrating its external, watertight sealed borders. Differently from HK-2 cells (Fig. 2H, I), ARPCs showed a distinctive morphology which evidenced their plasticity, namely a clearly elongated aspect and a partially oriented arrangement of actin filaments (Fig. 3H, I). This elongated configuration is often observed in ARPCs, even if they are differentiated in epithelial cells (Fig. 4A). In addition, the formation of junctions between adjacent ARPCs in a layer was visualized by ZO-1 immunostaining (Fig. 4B).

**Figure 5:**
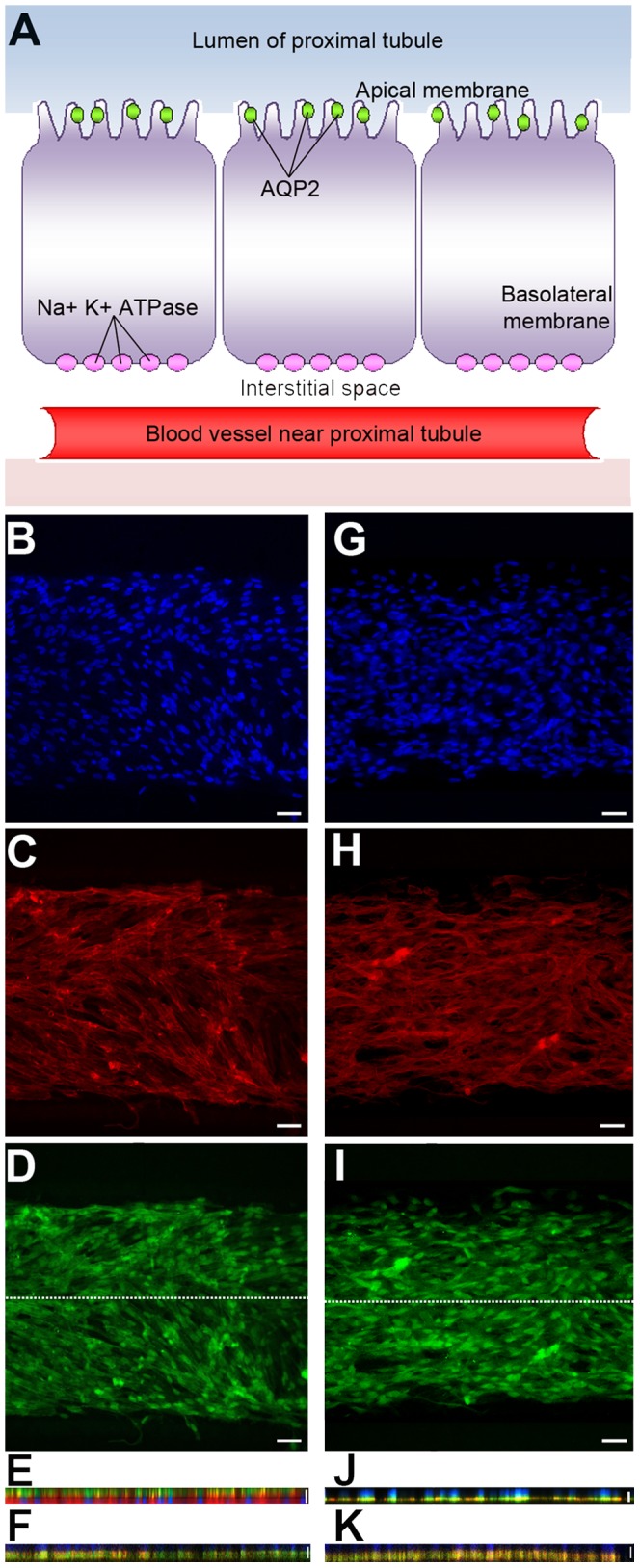
Polarization of ARPCs following FSS in the chip. (A) Cell model of proximal tubule cells with transporters. The scheme shows the AQP2 transporter localized in the apical membrane and the Na^+^K^+^ATPase present in the basolateral membrane. (B–J) Cellular arrangement of ARPCs grown into the microfluidic device after 6 hs of FSS at 0.2 dyn/cm^2^. Two different chips were stained in (B–E) and in (G–J), respectively. Immunofluorescence images of stained DNA (DAPI, blue) (B, G), actin (TRITC-phalloidin, red) (C, H), AQP2 (FITC-anti goat, green) (D) and Na^+^K^+^ATPase pump (I). *X*–*Z* section confocal images for AQP2 (apical marker protein) (E) and Na^+^K^+^ATPase pump (basolateral marker protein) (J) following FSS in the chip. Scale bars = 15 µm. The section along the longitudinal axis of the microchannel is indicated by the white line in D and I, respectively. *X*–*Z* section confocal images were also collected for AQP2 (F) and Na^+^K^+^ATPase pump (K) in statically cultured ARPCs. Scale bars = 15 µm.

Finally, the functional response of ARPCs was investigated, as induced by the exposure to the FSS (Fig. 5A). For this aim, a triple staining was carried out for visualizing nuclei (DAPI, Fig. 5B and 5G), actin filaments (phalloidin-TRITC, Fig. 5C and 5H), and the markers AQP2 or Na^+^K^+^ATPase pump (by their relative primary and secondary antibodies, green fluorescence), which are an apical and a basolateral protein, respectively (Fig. 5D and 5I). As shown in Fig. 5E, AQP2 was localized at the apical region of cells, whereas the Na^+^K^+^ATPase pump (Fig. 5J) was observed in the basolateral portion of cells, thus demonstrating a perfectly organized cell polarization. On the contrary, in statically cultured ARPCs, AQP2 (Fig. 5F) and Na^+^K^+^ATPase pump (Fig. 5K) did not localize at the extremities of cells but showed a diffuse cytoplasmic distribution, as typical of an unpolarized phenotype. The absence of a polarized morphology was also evidenced in macroscopic cultures, performed in standard two-compartment systems with static liquid conditions, as shown in the Fig. S3 of Supplementary Information.

## Discussion

The kidney is still one of the most difficult organs to be successfully mimicked and studied *in vitro*, since it presents a high structural and functional complexity, along with a distinctive cellular variety. The understanding of both physiological and pathological aspects of the different portions of the kidney may significantly benefit from the realization of miniaturized organ-on-chip devices, which combine biological and engineering approaches [11], [12], [29], [53].

In the present microsystem, the *in vivo* tubule-like environment was closely resembled, since the upper microchannel provided a lumen area, in which the apical portion of cells was exposed, while the lower microchannel simulated the interstitial area, in contact with the basolateral membranes of cells. Moreover, this geometry was suitable to induce the functional polarization of epithelial cell lines [20].

To mimic the characteristic kidney tubule microenvironment, it is important to consider the presence of the basement membrane, composed by several ECM proteins and acting as structural and functional meshwork for tubule epithelial and endothelial cells [44]. We found that fibronectin promoted an enhanced cell proliferation compared to other proteins. Consistently with this result, the positive reaction to fibronectin is considered a key phenotype indicator of well-differentiated proximal tubular cells [33], [54].

The next fundamental step to reproduce physiologically meaningful functions at device level was to understand the conditions by which renal tubule cells would cover to confluence the membrane surface. This is still one of the major drawbacks in the realization of a fully functional renal biochip [52]. In this work, the culture conditions on-chip were optimized by using HK-2 cells as model. We found that the confluence was achieved by seeding an initial concentration of 1.5×10^6^ cells mL^−1^ and letting cells grow under a static culture environment for about 24 h and then replacing the culture medium twice a day.

Once defined the optimal working conditions, we realized a functional biochip with renal tubule cells. After 4 days of cell culture, we perfused the lumen microchannel with the complete culture medium containing urea and creatinine and the interstitial microchannel with the same solution without urea and creatinine in a counter-current mode. Then, we collected the outlet samples from both lumen and interstitial microchannels to quantify the recovery of urea and creatinine. Creatinine and urea are ideal molecular probes since they are partially reabsorbed or secreted along the proximal tubule portion, therefore it is expected that the presence of a continuous layer of cells determines a variation of their permeability across the microporous membrane. Indeed, we obtained a lower recovery for urea and creatinine for devices with cells [(16±5)% and (18±5)%, respectively] with respect to the controls without cells [(64±7)% and (45±7)%]. This result clearly indicated a lower permeability in the presence of a highly uniform layer of HK-2 cells on the membrane surface, also confirmed by immunocytochemistry assays.

Previous bioartificial kidney devices have been realized by involving cell lines that grow easily in culture, such as cancer-porcine [51], canine [12], rat cells [11] and, among human cell lines, renal proximal tubular epithelial cells directly isolated [53], [55] or genetically modified to extend their lifespan [52], [44]. However, these devices are poorly predictive and lack the ability to reconstitute the real structural and functional features of human living kidney. Here we extracted ARPCs from the tubular portion of the cortex renal fraction and characterized them for renal stem cell markers (CD133, CD24, PAX-2, CD44, Oct-4, and BMI-1). Then, we cultured ARPCs in the device under the conditions previously optimized with HK-2 cells. After 4 days, we measured a recovery of urea and creatinine [(20±5)% and (13±5)%, respectively]. We also measured the recovery of glucose, which was found to be of (52±5)% in the ARPC-based device. This value could be further increased by exploiting more mature phenotypes of ARPCs compared to those used in this work, namely cells fully differentiated into epithelial cells and hence fully exhibiting transepithelial transport processes such as those involving glucose, or bicarbonate, and the associated net fluid reabsorption [56].

Finally, a polarization of ARPCs was induced by the FSS in the chip. AQP2 was localized at the apical region of cells, whereas the Na^+^K^+^ATPase pump was observed in the basolateral portion of cells, thus demonstrating a perfectly organized cell polarization. On the contrary, in statically cultured ARPCs, AQP2 and Na^+^K^+^ATPase pump did not localize at the extremities of cells but showed a diffuse distribution, as typical of an unpolarized phenotype.

Overall, the bioartificial proximal tubule-like, ARPC-embedding renal microdevice developed in this work provides an important proof of principle, since it integrates the topological, structural, chemical and biological features proper of the living kidney. On one hand, it provides a biomimetic platform to efficiently culture and analyze the physiological and pathological response of renal tubule cells. The culture of ARPCs, used for the first time in a miniaturized chip, demonstrates that it is possible to induce a well-defined polarization as highlighted by the apical and basolateral marker proteins. On the other hand, while this “*micro-organoid-on-a-chip*” device will need further characterization and validation, it opens new perspectives to recapitulate physiological functions. For this reason, the scale up and parallelization of such a single bioartificial renal tubule to multiple integrated microsystems, and ultimately its integration with other functional modules, might drive the development of new adjuvant for replacement therapy in kidney disease.

## Supporting Information

Figure S1 **Immunostaining of FN coating along the microchannel.** (A) The assay was performed directly inside the microchip, by using sequentially a primary anti-Fibronectin antibody and the secondary antibody labeled with Fluorescein Isothiocyanate. (B) shows the negative control represented by the staining of a device not functionalized with fibronectin.
(TIF)

Figure S2 **Investigation of RPTEC growth on-chip.** (A) Optical micrograph demonstrating the standard growth of RPTECs in a conventional polystyrene flask. (B) RPTECs unsuccessful growth into the microfluidic device. Scale bar: 100 µm.
(TIF)

Figure S3 **Polarization of ARPCs in a static macroscopic system.** *X-Z* section confocal images for AQP2 (apical marker protein) (A) and Na^+^K^+^ATPase pump (basolateral marker protein) (B) in ARPCs cultured in a culturing system with two compartments separated by the polycarbonate membrane and using static conditions of liquids.
(TIF)

Information S1 **Immunofluorescent staining of FN in the microchannel.**
(DOCX)
