# Correction: A Bioartificial Renal Tubule Device Embedding Human Renal Stem/Progenitor Cells

**DOI:** 10.1371/journal.pone.0100141

**Published:** 2014-06-06

**Authors:** The PLOS ONE Staff

The figure legends for Figure 2 and 5 are incorrect. There are incorrect values presented for the scale bars in the legends. Please see these figures and their corrected legends below.

**Figure 2:**
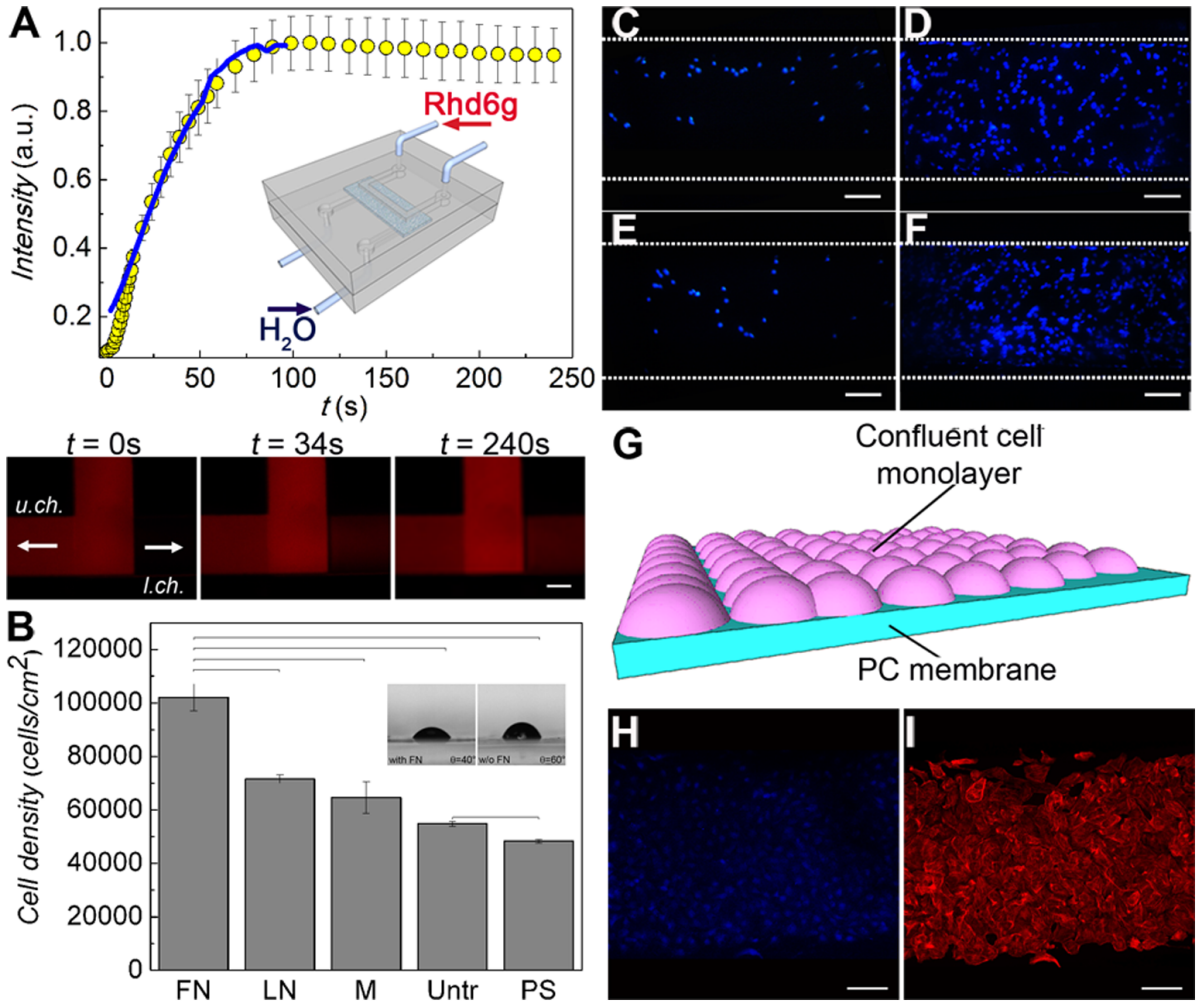
On-chip formation of a confluent monolayer of renal tubule cells. (A) Device fluidic characterization by diffusion test: a solution of rhodamine 6G was fed in the upper channel while distilled water was fed in the lower channel (interstitial). The increase in the fluorescence intensity with time at the outlet of the lower channel was correlated to the rhodamine diffusion into the channel. The superimposed line is the corresponding data in a standard cell culture insert using the same porous membrane. Inset: device and counter-current flow schematics. Scale bars  =  250 µm. (B) HK-2 cell proliferation described by MTS assay after 2 days of culture on the membranes functionalized with fibronectin (FN), laminin (LN) and Matrigel (M), compared to the untreated membranes (Untr.) and the positive controls of polystyrene dishes (PS). Results are expressed as (mean ± standard deviation) of three *independent experiments*. Bars show statistically significant differences (P<0.05). (*inset*) Optical micrographs of water droplets on membranes with or without FN, and corresponding WCA value. (C-F) Optimization of HK-2 cell growth in the device. A starting concentration of 5×10^5^ cells mL^-1^ was insufficient for a successful colonization of the membrane by cells (C), while a concentration ≥ 1.5×10^6^ cells mL^-1^ led to a confluent growth (D). Culturing cells for 4 days under a constant flux (1 µL min^-1^) of cell medium did not allow the formation of a continuous monolayer (E). Cell confluence was instead achieved by letting seeded cells in a static fluid environment for 24 h and culturing them over 4 days by changing the complete growth medium twice a day (F). Scale bars  =  100 µm. (G) Scheme of the resulting cell confluent monolayer on the membrane. Confluent living cells were stained with DAPI (blue) (H) and TRITC-phalloidin (red) (I). Scale bars  =  100 µm.

**Figure 5:**
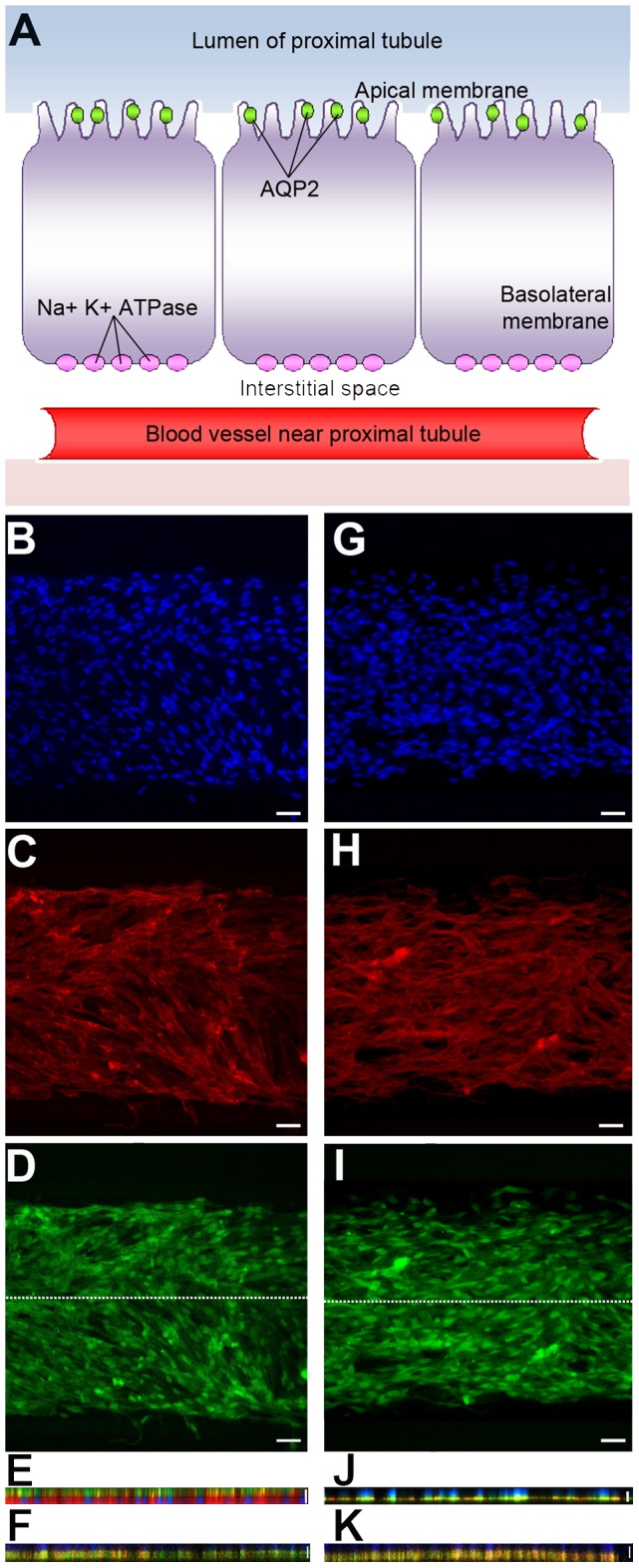
Polarization of ARPCs following FSS in the chip. (A) Cell model of proximal tubule cells with transporters. The scheme shows the AQP2 transporter localized in the apical membrane and the Na^+^K^+^ATPase present in the basolateral membrane. (B-J) Cellular arrangement of ARPCs grown into the microfluidic device after 6 hs of FSS at 0.2 dyn/cm^2^. Two different chips were stained in (B-E) and in (G-J), respectively. Immunofluorescence images of stained DNA (DAPI, blue) (B, G), actin (TRITC-phalloidin, red) (C, H), AQP2 (FITC-anti goat, green) (D) and Na^+^K^+^ATPase pump (I). *X*-*Z* section confocal images for AQP2 (apical marker protein) (E) and Na^+^K^+^ATPase pump (basolateral marker protein) (J) following FSS in the chip. Scale bars  =  100 µm. The section along the longitudinal axis of the microchannel is indicated by the white line in D and I, respectively. *X*-*Z* section confocal images were also collected for AQP2 (F) and Na^+^K^+^ATPase pump (K) in statically cultured ARPCs. Scale bars  =  15 µm.
