# Correction: A Bioartificial Renal Tubule Device Embedding Human Renal Stem/Progenitor Cells

**DOI:** 10.1371/journal.pone.0128261

**Published:** 2015-05-08

**Authors:** Anna Giovanna Sciancalepore, Fabio Sallustio, Salvatore Girardo, Laura Gioia Passione, Andrea Camposeo, Elisa Mele, Mirella Di Lorenzo, Vincenzo Costantino, Francesco Paolo Schena, Dario Pisignano

There are errors in Figure 3, panels E, F, and G. These panels were reproduced from Figure 1 of Sallustio F, Serino G, Costantino V, Curci C, Cox SN, et al. (2013) miR-1915 and miR-1225-5p Regulate the Expression of CD133, PAX2 and TLR2 in Adult Renal Progenitor Cells. PLOS ONE 8(7): e68296. doi:10.1371/journal.pone.0068296

Additionally, the cytofluorimetric images in Figure 3, panels B (CD133) and D (CD44) are the same respectively as Figure 1, panels A and C in Sallustio et al. PLOS ONE (2013). These panels represent the same cells in each article.

An investigation by a Scientific Committee at the University of Bari concluded that these image duplications arose by inadvertent error, due to poor management of the image archive, and that this does not affect the results and conclusions. The authors have provided the correct image files for Figure 3, panels E, F, and G in S1 File. A correction is also being made to Sallustio et al. PLOS ONE (2013).

**Fig 3. Fulfillment of a bioartificial proximal tubule on-a-chip embedding ARPCs.** (A) Scheme of the glomerulus and the proximal tubule structure in a human kidney nephron. Here tubular ARPCs were seeded into the device, whose cross section illustrates a confluent layer of ARPCs within the lumen microchannel and adherent to the membrane. After 4 days of culture, the lumen microchannel was perfused with the complete culture medium containing urea (UR) and creatinine (CR) and the medium without UR and CR was injected in counter-current into the lower, interstitial microchannel. (B–G) Characterization of isolated tubular ARPCs. Cytofluorimetric analysis shows the expression of CD133 (B), CD24 (C), CD44 (D). Immunofluorescence detection evidences the expression of Oct-4 (E), PAX-2 (F), BMI-1 (G). Scale bars = 50 μm. (H–I) Confluent growth of ARPCs in the device attested by immunostaining of cells with DAPI (blue) (H) and TRITC-phalloidin (red) (I). Scale bars = 100 μm.

## Supporting Information

S1 File Corrected files for Fig 3 E-G. Corrected files for Fig 3. Fulfillment of a bioartificial proximal tubule on-a-chip embedding ARPCs. Immunofluorescence detection evidences the expression of Oct-4 (E), PAX-2 (F), BMI-1 (G).
(ZIP)
