# Supplementary figures and images for: A Bioartificial Renal Tubule Device Embedding Human Renal Stem/Progenitor Cells
Source: PLoS One. 2014 Jan 30;9(1):e87496. doi: 10.1371/journal.pone.0087496 (PMC3907467; doi:10.1371/journal.pone.0087496)

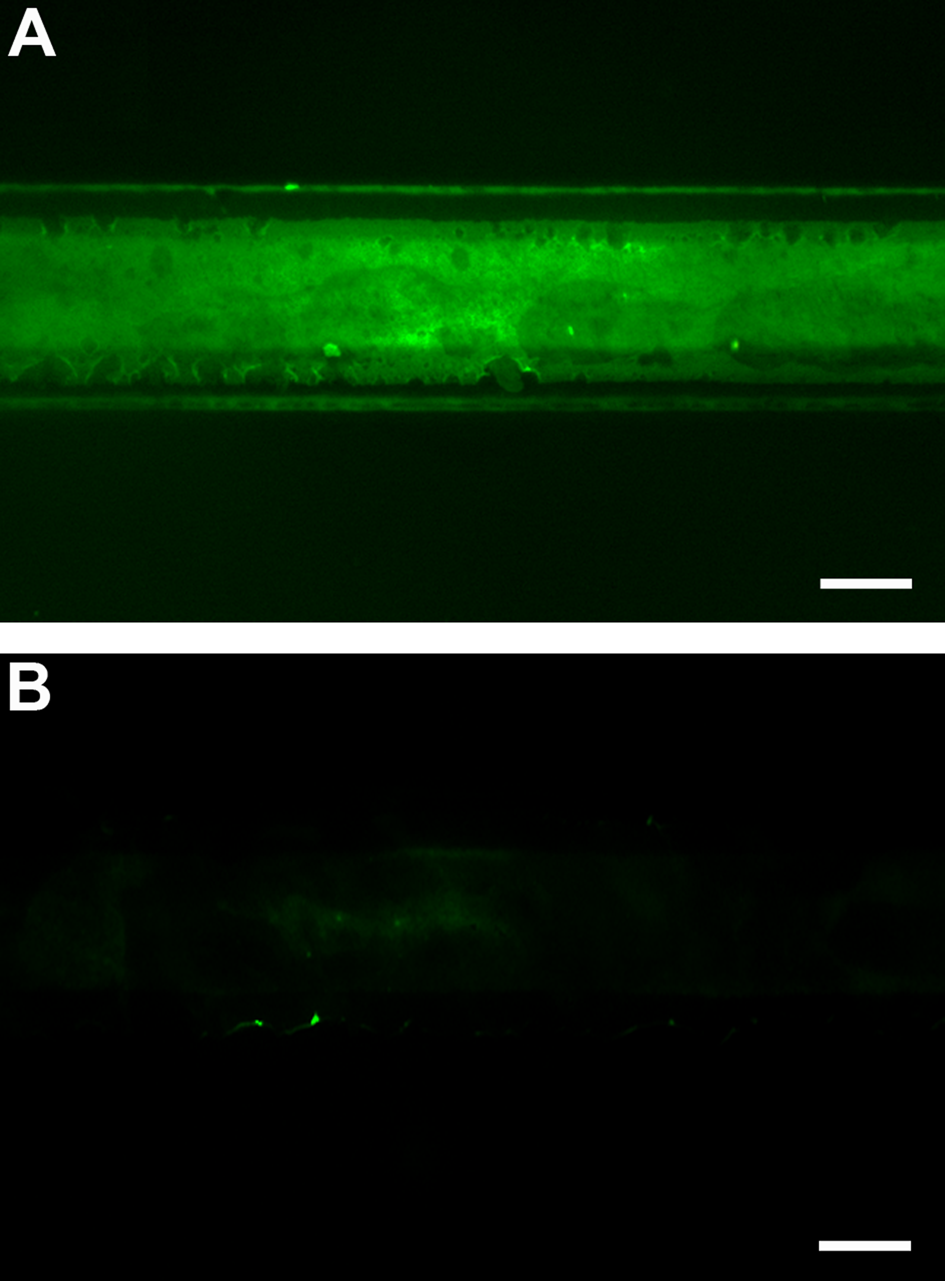

Supplement: Figure S1 — Immunostaining of FN coating along the microchannel. (A) The assay was performed directly inside the microchip, by using sequentially a primary anti-Fibronectin antibody and the secondary antibody labeled with Fluorescein Isothiocyanate. (B) shows the negative control represented by the staining of a device not functionalized with fibronectin. (TIF) [file pone.0087496.s001.tif]

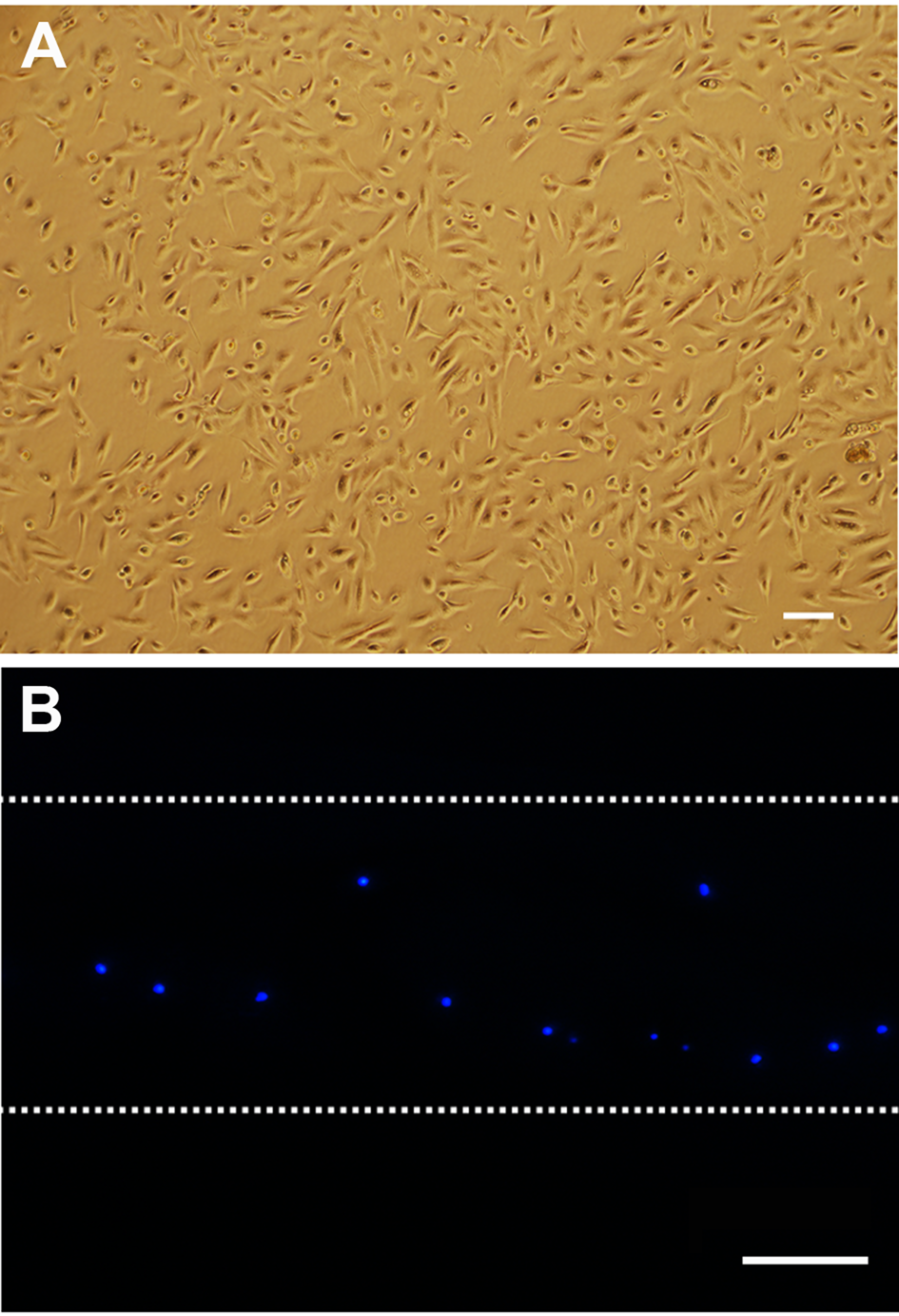

Supplement: Figure S2 — Investigation of RPTEC growth on-chip. (A) Optical micrograph demonstrating the standard growth of RPTECs in a conventional polystyrene flask. (B) RPTECs unsuccessful growth into the microfluidic device. Scale bar: 100 µm. (TIF) [file pone.0087496.s002.tif]

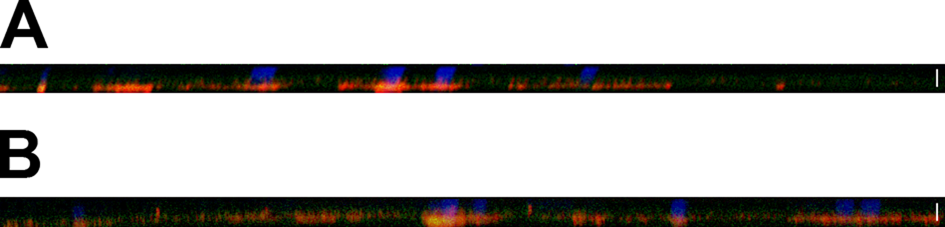

Supplement: Figure S3 — Polarization of ARPCs in a static macroscopic system. X-Z section confocal images for AQP2 (apical marker protein) (A) and Na+K+ATPase pump (basolateral marker protein) (B) in ARPCs cultured in a culturing system with two compartments separated by the polycarbonate membrane and using static conditions of liquids. (TIF) [file pone.0087496.s003.tif]
