# Supplementary material for: A Bioartificial Renal Tubule Device Embedding Human Renal Stem/Progenitor Cells
Source: PLoS One. 2014 Jan 30;9(1):e87496. doi: 10.1371/journal.pone.0087496 (PMC3907467; doi:10.1371/journal.pone.0087496)
Supplement: Information S1 — Immunofluorescent staining of FN in the microchannel. (DOCX) [file pone.0087496.s004.docx]

**A bioartificial renal tubule device embedding human**

**renal stem/progenitor cells**

Anna Giovanna Sciancalepore^a^, Fabio Sallustio^b,c,d^, Salvatore Girardo^e,£^, Laura Gioia Passione^a,f,e^, Andrea Camposeo^a,e^, Elisa Mele^a,§^, Mirella Di Lorenzo^e,#^, Vincenzo Costantino^b^, Francesco Paolo Schena^b,c^, Dario Pisignano^a,e,f^

^a^ Center for Biomolecular Nanotechnologies, Istituto Italiano di Tecnologia, Arnesano, Italy

^b^ Nephrology, Dialysis and Transplantation Unit, Department of Emergency and Organ Transplantation, University of Bari, Bari, Italy

^c^ Centro Addestramento Ricerca Scientifica in Oncologia (C.A.R.S.O.) Consortium, Valenzano, Italy

^d^ Department of Science, Biological and Environmental Sciences and Technologies, University of Salento, Lecce, Italy

^e^ National Nanotechnology Laboratory of Istituto Nanoscienze-CNR, Lecce, Italy

^f^ Dipartimento di Matematica e Fisica “Ennio De Giorgi”, Università del Salento, Lecce, Italy

^£^ Present address: Biotechnology Center, Technische Universität Dresden, Dresden, Germany

^§^ Present address: Nanophysics, Istituto Italiano di Tecnologia, Genoa, Italy

^#^ Present address: Department of Chemical Engineering, University of Bath, Bath, United Kingdom

Email: [anna.sciancalepore@iit.it](mailto:anna.sciancalepore@iit.it); [dario.pisignano@unisalento.it](file:///C:\Documents%20and%20Settings\asciancalepore\Local%20Settings\Temp\dario.pisignano@unisalento.it).

SUPPLEMENTARY INFORMATION

**Immunofluorescent staining of FN in the microchannel**

To investigate the distribution of the fibronectin coating along the microchannel, an immunofluorescent assay was performed. The polycarbonate membrane into the microfluidic device was covered with fibronectin (10 μg mL^-1^ in PBS) by a syringe-mediated introduction. After 2 h at 4°C, the device was rinsed with PBS and injected with bovine serum albumin (BSA, 1 % w/v) for 5 min to reduce nonspecific background staining. After washing with PBS, the chip was incubated for 5 min at room temperature with primary antibody (1:1000 rabbit anti-Fibronectin antibody in PBS). Then, the device was washed for 5 minutes in PBS solution and incubated for 5 min with secondary antibody (1:40, Fluorescein Isothiocyanate labelled anti-rabbit IgG antibody, in PBS). The device was further rinsed thrice in PBS for 5 min. A device without the fibronectin functionalization was used as negative control. After staining, the membranes were taken apart from the device and observed by stereomicroscope.
